# Influencing Factors and Molecular Pathogenesis of Sarcopenia and Osteosarcopenia in Chronic Liver Disease

**DOI:** 10.3390/life11090899

**Published:** 2021-08-30

**Authors:** Chisato Saeki, Akihito Tsubota

**Affiliations:** 1 Division of Gastroenterology and Hepatology, Department of Internal Medicine, The Jikei University School of Medicine, 3-25-8 Nishi-shimbashi, Minato-ku, Tokyo 105-8461, Japan; chisato@jikei.ac.jp; 2 Core Research Facilities, Research Center for Medical Science, The Jikei University School of Medicine, 3-25-8 Nishi-shimbashi, Minato-ku, Tokyo 105-8461, Japan

**Keywords:** chronic liver disease, sarcopenia, osteoporosis, osteosarcopenia, molecular mechanism

## Abstract

The liver plays a pivotal role in nutrient/energy metabolism and storage, anabolic hormone regulation, ammonia detoxification, and cytokine production. Impaired liver function can cause malnutrition, hyperammonemia, and chronic inflammation, leading to an imbalance between muscle protein synthesis and proteolysis. Patients with chronic liver disease (CLD) have a high prevalence of sarcopenia, characterized by progressive loss of muscle mass and function, affecting health-related quality of life and prognosis. Recent reports have revealed that osteosarcopenia, defined as the concomitant occurrence of sarcopenia and osteoporosis, is also highly prevalent in patients with CLD. Since the differentiation and growth of muscles and bones are closely interrelated through mechanical and biochemical communication, sarcopenia and osteoporosis often progress concurrently and affect each other. Osteosarcopenia further exacerbates unfavorable health outcomes, such as vertebral fracture and frailty. Therefore, a comprehensive assessment of sarcopenia, osteoporosis, and osteosarcopenia, and an understanding of the pathogenic mechanisms involving the liver, bones, and muscles, are important for prevention and treatment. This review summarizes the molecular mechanisms of sarcopenia and osteosarcopenia elucidated to data in hopes of promoting advances in treating these musculoskeletal disorders in patients with CLD.

## 1. Introduction

Sarcopenia is a syndrome characterized by decreased muscle mass and function (strength and/or physical performance) [1,2,3,4,5]. In 1989, Rosenberg proposed the concept of sarcopenia as the age-related loss of muscle mass [6]. Later research emphasized that loss of muscle function is important for diagnosing sarcopenia, which can occur regardless of age [1,2,3,4,5]. The European Working Group on Sarcopenia in Older People (EWGSOP) has classified sarcopenia into primary (when no other cause exists other than aging) and secondary (when the condition is caused by underlying diseases such as chronic liver disease (CLD)) [1]. The liver is a multifunctional organ involved in glucose and energy metabolism, hormonal regulation, cytokine production, and ammonia detoxification. Impairment of liver function can cause malnutrition, hyperammonemia, chronic inflammation, and imbalance of muscle protein synthesis and proteolysis, leading to sarcopenia [5,7]. Thus, sarcopenia is highly prevalent in patients with CLD, especially those with liver cirrhosis (LC) (30–70%) [8]. Notably, sarcopenia negatively affects health-related quality of life and prognosis, and increases the risk of complications, such as infection [9,10].

Several studies revealed a close relationship between sarcopenia and osteoporosis in community-dwelling older adults. This finding has fueled the concept of osteosarcopenia, defined as the concomitant occurrence of sarcopenia and osteoporosis [11,12,13,14]. These two musculoskeletal disorders affect each other and share a common genetic, mechanical, and biochemical pathophysiology [15,16,17,18]. Osteosarcopenia has been associated with more negative health outcomes than either sarcopenia or osteoporosis alone, increasing the risk of falls, fractures, and mortality [19]. Therefore, it has been described as a “hazardous duet” [19]. Our recent studies revealed that osteosarcopenia frequently develops and is associated with fractures, low physical performance, and frailty in patients with CLD [20,21,22]. Although sarcopenia and osteosarcopenia are a global health concern, appropriate assessment and early diagnosis of these musculoskeletal disorders in patients with CLD remain inadequate in real-world clinical settings. Furthermore, treatment strategies for osteosarcopenia in CLD have yet to be established, limiting our understanding of the pathogenic mechanisms involving the liver, bones, and muscles in patients with CLD. Therefore, this review summarizes the clinical significance and molecular mechanisms of sarcopenia and osteosarcopenia elucidated by clinical and basic research to date, in hopes of promoting advances in the treatment of musculoskeletal disorders in patients with CLD.

## 2. Materials and Methods Sarcopenia in Chronic Liver Disease

### 2.1. Assessment Criteria for Sarcopenia

The assessment criteria for sarcopenia have changed over time and vary among different working groups. Table 1 summarizes the reference values established by the EWGSOP [1,2], Asian Working Group for Sarcopenia (AWGS) [3,4], and Japan Society of Hepatology (JSH) [5]. In Europe, the EWGSOP initially published a definition for sarcopenia in 2010, subsequently revised in 2018 (EWGSOP2) based on accumulated scientific and clinical evidence [1,2]. EWGSOP2 particularly emphasizes low muscle strength as an important determinant of sarcopenia [2]. As a simple screening tool for sarcopenia, EWGSOP2 recommends the use of the self-reported SARC-F questionnaire consisting of the following five components: strength (S), assistance with walking (A), rising from a chair (R), climbing stairs (C), and falling (F). Each component is scored from 0 to 2, with a total score of ≥4 points suggesting suspected sarcopenia [2,23]. However, SARC-F has extremely low sensitivity (3.8–9.9%), although it has exceedingly high specificity (94–99%) [24]. Given the sheer size of Asia with its rapidly aging population, sarcopenia has a considerable impact within this region. Therefore, the AWGS established assessment criteria for sarcopenia among Asian populations in 2014, and revised the diagnostic algorithm and criteria in 2019 (AWGS 2019) [3,4]. According to the AWGS 2019, sarcopenia is defined as a loss of skeletal muscle mass and strength and/or reduced physical performance (e.g., gait speed <1.0 m/s) [4]. Similar to the EWGSOP2 criteria, severe sarcopenia is diagnosed when all three criteria are satisfied. As an initial screening for sarcopenia, the AWGS 2019 also recommends SARC-F and calf circumference measurement (cutoff values: <34 and <33 cm for men and women, respectively). Meanwhile, the JSH proposed simple and practical sarcopenia criteria for patients with CLD in 2016 [5], with the following characteristics: (1) omission of age restriction given that secondary sarcopenia in CLD is associated with nutritional and hormonal impairment as well as inflammatory cytokines rather than aging itself, (2) omission of gait speed assessment due to its complexity, and (3) the establishment of reference values for muscle mass using computed tomography (CT) given that patients with CLD undergo regular CT examinations [25]. Using CT, the skeletal muscle mass index (SMI) is calculated as the sum of muscle mass at the L3 level divided by the height squared, which is strongly correlated with SMI measured using the bioelectrical impedance analysis method [5]. Additionally, SMI calculated as the left–right sum of long axis × short axis of the iliopsoas muscles at the L3 level divided by the height squared, and psoas muscle index calculated as the left–right sum of the iliopsoas muscle area (with the manual trace method) divided by the height squared, could be adopted as alternative simple procedures [5,26]. CT also evaluates myosteatosis, which is characterized by the pathological accumulation of fat in skeletal muscle [27]. Myosteatosis is highly prevalent and associated with hepatic encephalopathy and mortality in patients with LC [28]. Therefore, CT could be a suitable and useful option for assessing muscle mass in patients with CLD.

In our study of 142 patients with LC, we compared the diagnostic performance of sarcopenia using the JSH, AWGS (gait speed included as an essential requirement), and EWGSOP2 criteria [20]. Accordingly, our results showed the diagnostic outcome of the JSH criteria was identical to that of the AWGS criteria (33.8% (48/142)), and similar to that of the EWGSOP2 criteria (28.2% (40/142)). These results suggest that patients with low muscle strength often have low gait speed, which may be more important for assessing the severity of sarcopenia rather than its diagnosis.

In general, the prevalence of sarcopenia increases with the progression of CLD stage [29]. However, a recent report showed that patients with alcoholic LC (ALC) were significantly younger and had a lower prevalence of sarcopenia than those with non-ALC despite demonstrating a more advanced disease stage [30]. Therefore, during sarcopenia analysis, patients with ALC may need to be treated separately from LC due to other etiologies.

### 2.2. Prevalence and Clinical Significance of Sarcopenia in Patients with Chronic Liver Disease

In general, age-related loss of skeletal muscle mass occurs at a rate of 1% per year up to 70 years of age and increases to 1.5% after that. However, among patients with LC, those categorized as Child–Pugh class A, B, and C have a rate of 1.3%, 3.5%, and 6.1%, respectively [31]. In large-scale studies involving Japanese older adults, the prevalence of sarcopenia ranged between 7.5% and 8.2% [32,33]. Our previous study, including 291 patients with CLD, reported a prevalence rate of 26.8% [22]. Another study on 142 patients with LC found a sarcopenia frequency of 33.8% [20]. These results suggest that patients with CLD, especially those with advanced liver disease, are more susceptible to skeletal muscle loss and sarcopenia compared to the general population. In patients with LC, older age, lower body mass index, and lower branched-chain amino acid (BCAA) and insulin-like growth factor 1 (IGF-1) levels were associated with sarcopenia [20]. Notably, osteoporosis was also associated with sarcopenia (odds ratio (OR) = 3.92), and vice versa (OR = 5.72), suggesting a close relationship between these two musculoskeletal disorders. Patients with sarcopenia have higher mortality rates than those without sarcopenia. BCAA supplementation improves the survival rates of sarcopenic patients with LC [10]. A cohort study on 631 patients with CLD revealed that low grip strength and calf circumference were independent factors associated with overall survival [34]. Moreover, a recent retrospective multicenter study involving 1,624 patients with CLD demonstrated that low muscle strength was an independent predictor of mortality. The cutoff values of handgrip strength for prognosis were 27.8 and 18.8 kg in men and women, respectively, similar to the reference values utilized in the AWGS criteria [35]. These results indicate that sarcopenia, especially with low muscle strength, is associated with mortality and is a useful predictor of prognosis in patients with CLD.

### 2.3. Pathogenic Mechanism of Sarcopenia

#### 2.3.1. Imbalance of Protein Anabolism and Catabolism

Skeletal muscle is the most major reservoir for protein. Under normal physiological conditions, skeletal muscle mass and protein turnover are maintained through a controlled balance between protein anabolism (synthesis) and catabolism (breakdown or proteolysis). Decreased protein synthesis and increased proteolysis cause muscle loss. Although sarcopenia is a common complication in patients with LC, it is caused by the interrelationship of multiple factors, such as attenuated protein anabolism due to impaired hepatic function, increased gluconeogenesis due to depleted glycogen reserves, excessive muscle protein breakdown, disease-related malnutrition, hyperammonemia due to impaired detoxification ability, reduced levels of anabolic hormones (e.g., IGF-1 and testosterone), and increased levels of inflammatory and catabolic cytokines [8,36,37,38,39,40,41,42].

#### 2.3.2. Suppressed Muscle Growth by Decreased IGF-1 Levels

**Figure 1:**
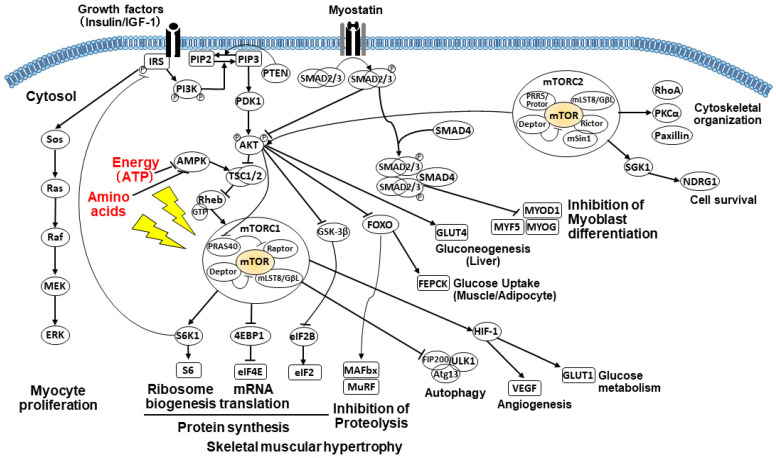
Involvement of the PI3K/AKT/mTOR signaling pathway in muscle protein synthesis and proteolysis. Selected abbreviations: AMP, adenosine monophosphate; AMPK, AMP-activated protein kinase; ATP, adenosine triphosphate; eIF, eukaryotic initiation factor; 4EBP1, 4E-binding protein 1; FOXO, forkhead box O; GSK3β, glycogen synthase kinase 3 beta; GTP, guanosine triphosphate; MAFbx, muscle atrophy F-box; mTOR, mechanistic target of rapamycin; mTORC1, mechanistic target of rapamycin kinase complex 1; MuRF1, muscle-specific RING-finger protein 1; PDK1, pyruvate dehydrogenase kinase 1; PI3K, phosphatidylinositol 3-kinase; PIP3, plasma membrane intrinsic protein 3; Rheb, Ras homolog enriched in brain; S6K1, S6 kinase 1; TSC1, tuberous sclerosis complex 1.

IGF-1 (also called somatomedin C) is produced by hepatocytes and myocytes. Its production is stimulated by growth hormones. IGF-1 has various paracrine, autocrine, and endocrine functions. The multimodal functions of IGF-I include the positive regulation of skeletal muscle growth [8,36,37,38,39,41]. IGF-1 binds to the IGF-1 receptor (IGF-1R), promoting the activation of phosphatidylinositol 3-kinase (PI3K). PI3K activation suppresses the function of tuberous sclerosis complex 1 (TSC1) and TSC2 (TSC1/TSC2 heterodimer) via a cascade of plasma membrane intrinsic protein 3 (PIP3), pyruvate dehydrogenase kinase 1 (PDK1), and AKT (protein kinase B) (Figure 1). TSC inhibition then increases the activity of guanosine triphosphate (GTP)-bound Ras homolog enriched in brain (Rheb), which directly activates the mechanistic target of rapamycin kinase complex 1 (mTORC1). Thus, IGF-1 activates mTORC1 signaling by suppressing TSC function via the PI3K-dependent pathway. The PI3K/AKT/mTOR pathway plays the most critical role in muscle protein anabolism. Target molecules downstream of mTORC1 regulate muscle protein synthesis and autophagic proteolysis for skeletal muscular hypertrophy.

Serum IGF-1 levels, which decrease with age and progression of liver disease stage, are especially low in patients with LC [39,43]. Low IGF-1 levels reduce mTOR activation during muscle protein synthesis, thereby increasing autophagic proteolysis [38,39]. IGF-1 is proposed to suppress myostatin gene expression via IGF-1/PI3K/AKT pathway activation [44]. Therefore, low IGF-1 levels can lead to increased myostatin expression. Such imbalanced conditions induced by decreased IGF-1 facilitate the development and further progression of sarcopenia. Moreover, a state of resistance to growth factors observed in LC accelerates a vicious cycle of reduced muscle protein synthesis and enhanced muscle breakdown.

#### 2.3.3. PI3K/AKT/mTOR Signaling Pathway

mTOR is a pleiotropic protein kinase that forms two distinct multicomponent complexes (mTORC1 and mTORC2). These mTOR complexes sense growth factors (e.g., insulin and IGF-1), nutrients (e.g., amino acids and energy), and cell stressors (e.g., hypoxia and toxicity) and regulate cell growth (increase in cell mass/size), proliferation, metabolism, protein synthesis, autophagy, and survival [36,37,40,45]. 

In skeletal muscle cells, mTORC1-mediated signaling promotes protein synthesis by activating the ribosomal protein S6 kinase 1 (S6K1) and inactivating the eukaryotic initiation factor (eIF) 4E-binding protein 1 (4EBP1) (Figure 1). S6K1, phosphorylated by mTORC1, enhances ribosome biogenesis and mRNA translation by activating 40S ribosomal protein S6 and other translation-related proteins. Non-phosphorylated 4EBP1 functions as a translational repressor of eIF4E, whereas mTORC1-mediated 4EBP1 phosphorylation initiates translation. Moreover, mTORC1 promotes lipogenesis by increasing peroxisome proliferator–activated receptor (PPAR) γ and sterol regulatory element-binding protein (SREBP) activities. Accordingly, mTORC1 drives cell growth by coordinately promoting both protein and lipid synthesis.

Meanwhile, mTORC2 demonstrates non-overlapping functions with mTORC1. Moreover, mTORC2 does not regulate protein synthesis and does not respond to the cellular nutrient/energy status. However, mTORC2 plays a key role in cell proliferation and survival via the activation of AKT.

#### 2.3.4. Impact of Amino Acids on the mTOR Pathway

The rate of protein synthesis is directly dependent on amino acid uptake. Amino acid sufficiency stimulates mTORC1 more directly compared to the aforementioned TSC-mediated pathway [36,37,45]. Amino acid depletion (particularly leucine and isoleucine or arginine) reduces mTORC1 signaling and the expression of mTORC1 substrates S6K1 and 4EBP1 [37,45]. Several GTPases, such as recombination activating genes (RAGs) and RAS-like proto-oncogene A (RalA), promote mTORC1 signaling partly via Rheb and mitogen-activated protein kinase kinase kinase kinase 3 (MAP4K3) in response to amino acid sufficiency [45]. This amino acid pathway dominates growth factor receptor signaling [37].

BCAAs (valine, leucine, and isoleucine) are essential amino acids that the body cannot produce and must be obtained from dietary protein. BCAAs are involved in the formation and maintenance of the skeletal muscle [46]. As such, BCAA insufficiency in chronic liver disease leads to skeletal muscle mass loss, regardless of age [43,47]. BCAA deficiency inhibits fatty acid synthesis, enhances fatty acid β-oxidation in the liver, and increases fat mobilization in white adipose tissue through the AMPK-mTOR-FOXO1 (forkhead box O1) pathway [46]. In patients with decompensated LC, excessive amino acid consumption for increased gluconeogenesis is primarily compensated by proteolysis in skeletal muscle [40,48]. This process produces both BCAAs and aromatic amino acids (AAAs: tyrosine, phenylalanine, and tryptophan). However, the localized branched-chain keto dehydrogenase catabolizes only BCAAs in skeletal muscle [8,40,46,48]. Consequently, BCAAs decrease while AAAs increase, causing a decrease in the Fischer ratio [40,48]. Hence, BCAA depletion reduces protein synthesis and turnover.

#### 2.3.5. Impact of Energy on the mTOR Pathway

Insufficient cellular energy attenuates mTORC1 signaling [37,40]. Elevation in the cellular AMP (adenosine monophosphate)/ATP (adenosine triphosphate) ratio, which reflects metabolic stressors that inhibit ATP production (e.g., hypoxia and glucose deprivation) or stimulate ATP consumption, activates AMP-activated protein kinase (AMPK), a sensor and master regulator of cellular energy metabolism [45]. Under energy stress conditions, AMPK activates TSC2 and represses mTORC1 signaling [45]. Various cell stressors, such as hypoxia and osmotic stress, also inhibit mTORC1 signaling by augmenting TSC function [49]. Declined mTORC1 signaling also facilitates macroautophagy, a degradative process that keeps cells alive under nutrient depletion by breaking down cell components into amino acids and other small molecules [37].

#### 2.3.6. Alterations of Glucose Metabolism and Gluconeogenesis

Patients with CLD (especially LC) have disease-related malnutrition, characterized by a combination of phenotypical (body weight loss, low body mass index, or reduced muscle mass) and etiological criteria (reduced intake/absorption, inflammation, or disease burden). They are predisposed to decreased glycogen storage, synthesis, and breakdown, promoting increased gluconeogenesis using amino acids derived from myoprotein breakdown and decreased skeletal muscle mass [40,41]. Such alterations change energy metabolism from carbohydrate oxidation to fat oxidation, causing insulin resistance in the liver, skeletal muscle, and fat tissues [41,48]. In insulin resistance conditions, the impaired insulin receptor reduces the AKT/mTOR pathway activity, causing reduced protein synthesis, enhanced protein breakdown, and muscle atrophy through autophagy and the ubiquitin-proteasome system (UPS) [50]. In patients with LC who are predisposed to fasting/starvation states, proteolysis and protein turnover accelerate in skeletal muscle to efficiently yield enough glucose, resulting in additional amino acid consumption and depletion of muscle protein storage (i.e., skeletal muscle loss and sarcopenia) [40,48].

#### 2.3.7. Suppressed Muscle Growth by Elevated Myostatin and Increased Autophagy

Myostatin, a transforming growth factor beta (TGF-β) superfamily cytokine, is one of several myokines produced and released by skeletal muscle cells, and serves as a key inhibitor of skeletal muscle growth (i.e., hypertrophy and hyperplasia) [8,42]. IGF-1 and testosterone suppress myostatin, as described above [38,39,44] and hereinafter [51,52]. In patients with LC, decreased levels of IGF-1 and testosterone promote increased myostatin expression, reduced protein synthesis, and enhanced protein breakdown [8,40].

Myostatin signaling phosphorylates the SMAD2/3 complex via the activin receptor type IIB (ActRIIB), ubiquitously expressed on skeletal muscle cells [53]. The activated SMAD2/3 complex inhibits the phosphorylation of FOXO by suppressing AKT, thereby retaining FOXO in the myocyte nucleus. Otherwise, FOXO is phosphorylated by AKT, transported out of the nucleus, and degraded in the cytoplasm. The intranuclear FOXO regulates the transcription of target genes, facilitating autophagy and protein degradation via the UPS and inhibiting protein synthesis and cell proliferation/differentiation [36,53,54], thereby causing growth inhibition and skeletal muscle atrophy. Moreover, FOXO upregulates gluconeogenesis and lipid uptake (*LPL*), and downregulates lipogenesis (*SREBP1c*) and glycolysis (*PDK4*). Meanwhile, the SMAD2/3 complex-suppressed AKT/mTOR signaling pathway promotes the inhibition of skeletal muscle growth. The activation of SMAD pathway signaling inhibits the proliferation of satellite cells (tissue stem cells) and differentiation of myoblasts into myotubes by suppressing MYOD1, MYF5, and MYOG in a SMAD3-dependent manner [55].

In patients with LC, excessive muscle autophagy is mediated by hyperammonemia and oxidative stress or promoted via the upregulated UPS due to increased levels of proinflammatory cytokines (e.g., tumor necrosis factor-α (TNF-α) and interleukin-6 (IL-6)), which can cause skeletal muscle loss and sarcopenia [36,40,53,56,57]. Muscle autophagy is a constitutive system in which autophagosomes form and fuse with lysosomes to degrade cytoplasmic components (e.g., proteins and organelles), induced and maintained by FOXO members and suppressed by mTOR [58]. The overactive UPS is characterized by increased muscle-specific E3 ubiquitin ligases, such as muscle atrophy F-box (MAFbx; also called atrogin-1) and muscle-specific RING-finger protein 1 (MuRF1). It is one of the major catabolic mechanisms that cause the loss of skeletal muscle mass and sarcopenia [36,57]. Myostatin may promote inflammatory cytokine production (e.g., TNF-α) in muscle, insulin resistance in the muscle and liver, and liver steatosis [59]. Moreover, myostatin acts directly and suppressively on bone formation and regeneration [60].

#### 2.3.8. Impaired Molecular Functions via Hyperammonemia

Ammonia is normally detoxified via conversion into urea through the urea cycle in the liver. It can also be alternatively detoxified using glutamine synthetase in the skeletal muscle. In patients with LC, the skeletal muscle compensates for the decreased hepatic ability to detoxify ammonia. Therefore, hyperammonemia occurs when ammonia production exceeds the skeletal muscle’s and liver’s detoxification capacity. Hyperammonemia increases the expression of myostatin by activating nuclear factor kappa B (NF-κB) [40,61]. In individuals experiencing weight loss, serum and intramuscular myostatin levels increase and correlate inversely with skeletal muscle mass [62]. Elevated plasma myostatin levels are suggested to reduce skeletal muscle mass among patients with LC who have hyperammonemia [56]. Ammonia accumulation in skeletal muscle reduces α-ketoglutarate, an intermediate substrate of the tricarboxylic acid (TCA) cycle that can inhibit autophagy [63], causing the impairment of the TCA cycle, mitochondrial function, and ATP synthesis. Energy shortage (low ATP levels) can attenuate protein synthesis, given that it is an energy-intense process [61]. Moreover, hyperammonemia is involved in decreased ribosomal biogenesis [42,64] and increased autophagy [56]. Accordingly, hyperammonemia facilitates the development of sarcopenia through the liver–muscle axis [42,61]. In turn, patients with sarcopenia are predisposed to hyperammonemia and hepatic encephalopathy.

#### 2.3.9. Impact of Exercise on Muscle Anabolism

Physical activity and exercise are critical determinants of muscle anabolism, particularly in patients with LC [40]. Regular physical activity/exercise, including supervised physical training, effectively prevents and improves sarcopenia and its complications (e.g., frailty and osteosarcopenia). Aerobic and resistance exercise attenuates myostatin mRNA expression in skeletal muscle and serum myostatin levels [65,66]. Resistance exercise prevents skeletal muscle breakdown and deterioration of physical function while promoting increased muscle mass by upregulation of anabolic hormones (e.g., testosterone, IGF-1, and growth hormone) and activating the mTORC1 signaling pathway [36,40,45,67,68]. However, the optimal exercise programs for LC remain undetermined, and the underlying mechanisms have yet to be fully elucidated. Additionally, training programs should include regimens for protein and carbohydrate supplementation.

#### 2.3.10. Decreased Anabolic Hormone Testosterone

Testosterone is an anabolic–androgenic steroid hormone synthesized from cholesterol, mainly in the gonad and adrenal gland [67,68]. Circulating testosterone enters skeletal muscle by binding to membrane-bound or cytoplasmic androgen receptors (ARs). The testosterone/AR complex transforms, dimerizes, and translocates to the nucleus, which binds to specific androgen-response elements (AREs) on the DNA. These activated AREs stimulate the transcription of anabolic gene targets (e.g., IGF-1 and testosterone), promoting an increase in muscle protein synthesis, activation/proliferation/differentiation (into skeletal myocytes) of satellite cells and myoblasts, and hypertrophy [51,67,68,69]. Alternatively, testosterone activates the AKT/mTOR/S6K1 pathway through PI3K activation (Figure 1) [69,70,71] or stimulates G protein-linked membrane receptors, which activates Ras/ERK1/2 pathway signaling in a calcium-dependent manner, facilitating the phosphorylation of cell growth-related transcription factors [72]. Testosterone may also increase myogenesis through Notch signaling by suppressing myostatin and activating AKT signaling [52]. Androgens may increase follistatin (an antagonist to myostatin) in skeletal muscle satellite cells, thereby decreasing myostatin signaling molecules [51].

LC disturbs the hypothalamic–pituitary–gonadal axis, causing hypogonadism (low testosterone), although the mechanism has yet to be fully understood and is probably multifactorial [8,73,74]. Patients with LC frequently have low testosterone and high estrogen levels, clinically manifesting as palmar erythema, vascular spider, gynecomastia, testicular atrophy, and diminished libido. Testosterone deficiency is associated with sarcopenia, osteopenia, a shorter period to liver failure, and higher scores for hepatic reserve function (e.g., model for end-stage liver disease (MELD) score and the Child–Pugh–Turcotte (CPT) score), causing frailty and increased morbidity and mortality [73,74]. Testosterone supplementation improves serum testosterone levels, skeletal muscle mass and strength, bone mineral density, and frailty. However, it appears to exert no significant effects on bilirubin, coagulation profile, or creatinine except for albumin. At present, no conclusive data have been available on improving the aforementioned liver-related scores, complications (e.g., ascites and hepatic encephalopathy), readmission rates, or death.

#### 2.3.11. Other Factors Influencing Sarcopenia

Nutrition- and inflammation-related disorders are caused by prolonged acute and chronic diseases and lack of nutrient intake and/or absorption, promoting compromised body composition and function (so-called “disease-related malnutrition”) [40,41,48,75]. Malnutrition in LC is characterized by loss of skeletal muscle mass and function [8,40,48]. Reduced dietary and energy intake are principal causes of sarcopenia and attributable to various factors: neuroendocrine dysregulation of satiety and appetite (e.g., ghrelin, leptin, and proinflammatory cytokines), taste and olfactory disturbance, and malabsorption [8,75,76,77]. Ghrelin, a gastrointestinal hormone with orexigenic effects [78], might reduce the fibrogenic response of hepatic stellate cells, oxidative stress-/inflammation-induced liver injury, and myofibroblast accumulation, reducing liver fibrosis [79]. Patients with LC have lower ghrelin, higher postprandial glucose, and increased leptin levels [76,77,79]. Leptin, an anorexigenic hormone, inhibits orexigenic neurons and activates anorexigenic neurons in the hypothalamus [78]. Patients with LC who have portal hypertension and dysbiosis, which compromise the gut barrier function and cause the translocation of bacterial products, are in an activated proinflammatory state [40]. Such patients have increased proinflammatory cytokines, such as TNF-α, IL-1β, and IL-6, which are anorexic mediators and cause anorexia [41].

Dysgeusia and hyposmia are frequently noted in advanced liver diseases and may diminish nutrient intake, promoting nutrient deficiency. Zinc insufficiency or deficiency has been associated with altered senses of taste and smell as well as dysorexia [80]. Malabsorption has also been frequently caused by cholestasis, portal hypertension, and drug-related diarrhea (e.g., lactulose and antibiotics), causing malnutrition and sarcopenia in patients with LC [40,48,75].

## 3. Osteosarcopenia in Chronic Liver Disease

### 3.1. Prevalence and Clinical Significance of Osteosarcopenia in Patients with Chronic Liver Disease

In 2009, Binkley and Buehring advocated the concept of sarco-osteoporosis, which was then defined as the concomitant occurrence of sarcopenia and osteoporosis and has now evolved into the term “osteosarcopenia” [14]. Patients with osteosarcopenia have an increased risk of falls and fractures, resulting in a poor quality of life and increased mortality [81,82]. The prevalence of osteosarcopenia in community-dwelling older adults is 8.4 %, 12.7%, and 19.2 % in Japan, China, and Korea, respectively [11,12,13]. In a Korean study of patients aged ≥60 years with hip fractures, the prevalence of osteosarcopenia was 28.7%, and the 1-year mortality of osteosarcopenia (15.1%) was higher than that of the normal (7.8%), osteoporosis-alone (5.1%), and sarcopenia-alone (10.3%) groups [83]. Furthermore, patients with osteosarcopenia had higher scores for disability, frailty, and depression than those without it [13]. A recent pooled analysis of the aging general population demonstrated that osteosarcopenia increases the risk of fractures [odds ratio (OR), 2.46], falls (OR, 1.62), and mortality (OR, 1.66) [84].

As shown in Table 2, several studies have investigated the prevalence and clinical significance of osteosarcopenia in patients with CLD [20,21,22,85,86]. In one study of 142 patients with LC, the proportion of patients in the normal, sarcopenia-alone, osteoporosis-alone, and osteosarcopenia groups was 53.5%, 12.0%, 12.7%, and 21.8%, respectively [20]. In the osteosarcopenia group, the values of the skeletal muscle mass index (SMI) and handgrip strength were the lowest, whereas the prevalence of vertebral fractures was the highest (61.3%) among all four groups [20]. In another study of 117 patients with primary biliary cholangitis (PBC), the prevalence of osteosarcopenia was 15.4% [21]. Patients with osteosarcopenia had a higher prevalence of vertebral fractures than those without osteoporosis and sarcopenia (55.6% vs. 6.7%) [21]. In the other study of 291 patients with CLD, 49 (16.8%) and 81 (27.8%) had osteosarcopenia and frailty, respectively [22]. Frailty and vertebral fractures more frequently occurred in patients with osteosarcopenia than in those without (79.6% vs. 17.4% and 59.2% vs. 20.2%, respectively) [22]. Patients with osteosarcopenia also showed a greater impairment of physical performance and balance than those without osteosarcopenia, resulting in an increased risk of falls and fractures [87]. Furthermore, vertebral and hip fractures can cause impaired physical function and immobility, thereby leading to sarcopenia [88,89]. These findings suggest that osteosarcopenia and fractures are closely interrelated and exacerbate the negative health outcomes of each other.

### 3.2. Pathogenic Mechanisms of Osteosarcopenia: Relationship between Muscle and Bone

Given that muscles and bones are closely related during their development and growth, it is conceivable that sarcopenia, osteoporosis, and osteosarcopenia often progress in conjunction with each other [15,16,17,18]. Therefore, an understanding of the relationship between muscles and bones along with the underlying pathogenesis of osteosarcopenia is essential from a therapeutic point of view. Although the pathogenesis of osteosarcopenia in CLD is not fully elucidated, we addressed the possible mechanisms herein.

#### 3.2.1. Mechanical Factors

Increasing the mechanical load on the skeletal muscles leads to protein synthesis and muscle hypertrophy, while the opposite causes muscle atrophy [90]. The maintenance of bone mass and strength depends on the contribution of the following skeletal muscle-derived mechanical forces: (1) the tensile forces generated by contracting muscles at their insertion site; (2) the compressive forces between bones generated by muscles contracting through joints; and (3) the bending forces that long bones receive when the muscles generate the force to lift the object held distally [90]. The expression level of IGF-1, which has a positive effect on muscles and bones, is increased by exercise-induced mechanical loading [17]. Accordingly, reduced physical activity cannot maintain skeletal muscle and bone mass, resulting in the development and progression of sarcopenia and osteoporosis.

#### 3.2.2. Genetic Factors

During embryogenesis, muscles and bones originate from a common mesenchymal precursor, and their development is controlled by common genes and growth factors [91]. Therefore, genetic factors may influence both sarcopenia and osteoporosis. A genome-wide association study (GWAS) of Han Chinese and US Caucasians revealed that three single nucleotide polymorphisms in or near the glycine-N-acyltransferase (*GLYAT*) gene, which is essential for the regulation of glucose and energy metabolism, were associated with bone size and muscle mass [92]. A subsequent GWAS in the US identified *METTL21C* as a pleiotropic gene for bones and muscles [93]. *METTL21C* is highly expressed in muscles and plays an important role in myoblastic differentiation, calcium homeostasis, and survival of osteocytes against apoptosis through the modulation of NF-κB signaling [17,93]. In addition, myocyte enhancer factor 2C (*MEF-2C*) and α-actinin 3 (*ACTN3*) are candidate genes with a pleotropic effect on bones and muscles [94]. MEF-2C is a transcriptional regulatory protein involved in skeletal muscle development, sarcomeric gene expression, and fiber-type control; loss of MEF-2C results in disorganized myofibers [95]. MEF-2C also regulates bone homeostasis by modulating osteoclastic bone resorption, and deletion of *MEF-2C* results in increased bone mass [96]. *ACTN3* is highly expressed in fast glycolytic muscle fibers and contributes to the differentiation of muscle fibers toward the fast-twitch type [97]. Additionally, *ACTN3* is expressed in osteoblasts, and the deletion of *ACTN3* reduces bone mass [98].

#### 3.2.3. Chronic Inflammation

The production of reactive oxygen species (ROS) and proinflammatory cytokines, such as IL-1β, IL-6, and TNF-α, is increased in chronic disease conditions, including CLD [99,100]. Chronic inflammation inhibits protein synthesis and osteoblast differentiation and promotes protein breakdown and osteoclastic bone resorption, which lead to skeletal muscle and bone mass loss [5,17]. Advanced glycation end products (AGEs), which are induced by non-enzymatic glycation, oxidation, and chronic inflammation, suppress the expression of myogenic genes and impair the osteoblasts’ function, thereby affecting the quality of bone produced [17,101]. Pentosidine is one of the major AGEs and its well-characterized cross-link has been studied in bone tissues. The levels of pentosidine increases with age, and high levels of pentosidine are a risk factor for fractures in older adults. Serum pentosidine levels were reported to be negatively correlated with skeletal muscle mass in postmenopausal women with diabetes [102]. Notably, plasma pentosidine levels were increased in patients with a decreased liver functional reserve as well as in those with prevalent fractures [103]. Similar to other pathological conditions, CLD (especially LC) also facilitates a reduction in both skeletal muscle mass and bone mass and quality.

#### 3.2.4. Myokines

Skeletal muscle cells secrete various endocrine molecules, such as IGF-1, myostatin, irisin, beta-aminoisobutyric acid (BAIBA), fibroblast growth factor 2 (FGF2), IL-6, IL-7, IL-15, and osteoglycin, which influence bone metabolism [15,16,17,18]. IGF-1 is synthesized primarily in the liver and is also produced by muscles and bones [17]. As described above, IGF-1 regulates skeletal muscle protein synthesis via the PI3K/AKT/mTOR pathway. Moreover, IGF-1 stimulates osteoblast proliferation and contributes to the maintenance of bone mass and strength [104,105]. When CLD progresses to the advanced stages, serum IGF-1 levels decrease, leading to a loss of skeletal muscle and bone mass [44,105]. As described above, myostatin acts as a negative regulator of muscle cell proliferation and protein synthesis and is inhibited by IGF-1 and testosterone. Serum myostatin levels have not only been revealed to be higher in patients with decompensated LC than in those with compensated LC and healthy controls, but have also been associated with muscle mass loss and worse survival [106,107]. Notably, myostatin also negatively regulates bone formation and metabolism by promoting osteoclast differentiation [108]. Inhibition of the myostatin pathway results in an increase in not only muscle mass but also bone mass [109]. Irisin, a hormone-like myokine produced by skeletal muscles in response to physical exercise, is released into the circulation by cleavage of the fibronectin type Ⅲ domain-containing protein 5 (FNDC5) and plays a crucial role in the regulation of bone metabolism [110,111]. Administration of recombinant irisin increases cortical bone mass and strength and promotes pro-osteoblastic genes and osteoblastic bone formation, and it also reduces the effect of osteoblast inhibitors [112]. Intriguingly, irisin is highly expressed in hepatocytes, Kupffer cells, and sinusoidal endothelial cells in the human liver [113]. This suggests that CLD may decrease irisin levels that may subsequently affect bone mass and strength. Among patients with LC, it was revealed that not only were the serum irisin levels lower in sarcopenic patients than in non-sarcopenic patients, but also associated with sarcopenia [111]. FNDC5 deficiency impairs autophagy and fatty acid oxidation and enhances lipogenesis in the liver via the AMPK/mTOR pathway [114]. BAIBA, a small molecule secreted by skeletal muscles during exercise, protects osteocytes against ROS-induced apoptosis and prevents bone loss [115].

#### 3.2.5. Osteokines

As bones secrete various substances that affect other organs, they may also be considered as endocrine organs. Bones secrete various osteokines such as osteocalcin, sclerostin, Wnt, TGF-β, fibroblast growth factor 23 (FGF23), and prostaglandin E2 (PGE2), which affect the metabolism of not only bones but also muscles [15,16,17,18]. Osteocalcin is a noncollagenous protein secreted from osteoblasts [116]. Osteocalcin-deficient (Ocn^−/−^) mice showed reduced muscle mass, and treatment with exogenous osteocalcin increased muscle mass in older mice, whereas deletion of osteocalcin resulted in increased bone mass [117,118]. In contrast, another study using newly generated Ocn^−/−^ mice demonstrated that osteocalcin was not involved in the regulation of bone quantity and muscle mass [119]. Therefore, the impact of osteocalcin on muscles and bones remains controversial. The Wnt/β-catenin signaling pathway promotes osteoblast differentiation and osteogenesis and is involved in prenatal myogenesis and skeletal muscle regeneration/fibrosis [120,121]. Sclerostin, a protein encoded by the Sost gene and produced by mature osteocytes, acts as a negative regulator of the Wnt/β-catenin pathway, and therefore inhibits bone formation [122]. In patients with PBC, sclerostin was found to be expressed in the bile duct epithelium and was associated with the severity of cholangitis. In addition, the serum sclerostin levels in patients with PBC were higher than those in controls, suggesting its potential involvement in impaired bone formation [123]. A Korean study of healthy non-diabetic subjects showed that serum sclerostin levels were negatively correlated with skeletal muscle mass [124]. However, sclerostin-deficient mice showed greater trabecular bone volume and lower muscle mass than did wild-type mice [125]. Therefore, further studies are needed to clarify the role of sclerostin in the muscle–bone relationship. TGF-β, which is produced by osteoblasts and involved in the regulation of bone remodeling and homeostasis [126], induces muscle fiber atrophy by upregulating the E3 ubiquitin ligase atrogin-1 in mice [127]. In a mouse model of osteolytic bone metastases, bone-derived TGF-β contributed to muscle weakness by decreasing Ca^2+^-induced muscle force production [128].

#### 3.2.6. Vitamin D

The liver plays a crucial role in vitamin D metabolism. When the liver’s function is impaired, bone and muscle homeostases are dysregulated through vitamin D metabolism [129,130]. Vitamin D is not only involved in the intestinal absorption of calcium and phosphate and maintenance of appropriate circulating concentrations of these minerals, but also contributes to normal bone mineralization [129,130]. Vitamin D deficiency causes secondary hyperparathyroidism, leading to an increased bone turnover and consequent bone loss. An in vitro study of C_2_C_12_ skeletal muscle cells reported that vitamin D promoted the differentiation of myogenic cells by increasing the expression and nuclear translocation of the vitamin D receptor (VDR) and modulating promyogenic and antimyogenic factors [131]. VDR knockout and vitamin D-deficient mice showed decreased muscle mass and strength, dysregulation of myogenic regulatory factors, and increased myostatin and MuRF1 [132]. Reportedly, the rate of patients with CLD who frequently exhibited vitamin D deficiency (≤20 ng/mL) ranged from 47% to 87% [133]. In one study of patients with CLD, serum vitamin D levels were positively correlated with skeletal muscle mass and handgrip strength, and low vitamin D levels were associated with sarcopenia [134]. Our study revealed that low vitamin D levels, especially severe vitamin D deficiency (≤10.5 ng/mL), were closely related to sarcopenia and frailty in patients with CLD [133]. These findings suggest that maintaining sufficient levels of vitamin D is important for preventing loss of skeletal muscle and bone mass.

### 3.3. Treatment for Osteosarcopenia

#### 3.3.1. Physical Exercise

Optimal physical exercise has positive effects on skeletal muscle and bone mass. In one study of community-dwelling older adults with osteopenia or increased fall risk, a 12-month supervised, multicomponent exercise program consisting of progressive resistance, weight-bearing impact, and balance training thrice a week improved the bone mineral density (BMD), muscle strength, and physical function of the patients [135]. In another study of older men with osteosarcopenia, a 12-month high-intensity resistance training with protein, vitamin D, and calcium supplementation increased the SMI and maintained lumbar spine BMD, whereas these parameters were decreased in the control group [136]. Similarly, in patients with compensated LC, a 3-month moderate exercise program consisting mainly of cycle ergometry and treadmill walking three times per week increased lean body mass, lean appendicular mass, and lean leg mass and decreased fat body mass, with an improvement in physical performance as assessed by the Time Up and Go Test (TUG test) [137]. In patients with LC (Child–Pugh A/B), an 8-week home-based exercise training program of moderate to high-intensity cycling exercise thrice a week increased the 6-min walk distance and thigh muscle thickness [138]. These results suggest that physical exercise is effective in the treatment of osteosarcopenia; however, patients with decompensated LC, especially those with end-stage liver disease, should be aware of the risk of complications such as variceal bleeding [139].

#### 3.3.2. Nutritional Interventions

As described above, BCAAs activate the mTOR pathway for the proliferation of satellite cells, thereby promoting muscle protein synthesis and growth. A systematic review revealed that administration of leucine or leucine-enriched proteins mainly increases lean muscle mass, thereby improving sarcopenia in older adults [140]. In patients with LC, BCAA supplementation improved glucose metabolism and preserved skeletal muscle mass at 48 weeks, with an amelioration of hypoalbuminemia [141]. In patients with alcoholic LC, leucine-enriched BCAA supplementation restored the impaired mTOR signaling and reduced the muscle breakdown [142]. L-carnitine is involved in fatty acid and energy metabolism, and can either be obtained from diet or synthesized from the essential amino acids lysine and methionine in the liver and kidney [143,144,145]. In patients with LC, especially those with sarcopenia and malnutrition, carnitine deficiency is a frequent complication [145]. Patients with LC supplemented with L-carnitine had less skeletal muscle loss than those without treatment, despite having worse liver function [143]. Furthermore, administration of high-dose L-carnitine decreased serum ammonia levels and increased skeletal muscle mass [144]. Although numerous in vivo and in vitro studies have demonstrated the positive effects of vitamin D on both skeletal muscle and bone growth, its efficacy as a supplement for osteoporosis and sarcopenia remains controversial. A meta-analysis of 23 studies demonstrated that vitamin D supplementation slightly improved the BMD of the femoral neck, but not of the total hip [146]. Another meta-analysis of 81 randomized controlled trials (RCTs) revealed that vitamin D supplementation had no beneficial effect on the BMD or the prevention of fractures and falls [147]. In an RCT of postmenopausal women, the beneficial effects of vitamin D supplementation on the spine and hip BMD were evident in subjects with a baseline 25(OH) D ≤ 30 nmol/L [148]. Similarly, another RCT of community-dwelling older adults revealed that high-dose vitamin D supplementation had a significant effect on the BMD in subjects with a baseline 25(OH) D ≤ 30 nmol/L [149]. Regarding the effects on the muscles, 4-month supplementation of vitamin D increased the intramyonuclear VDR concentrations and muscle fiber size in older women with moderately low vitamin D status [150]. A meta-analysis of 29 RCTs revealed that vitamin D supplementation marginally improved handgrip strength but did not improve mobility as assessed by the TUG test [151]. The subgroup analyses demonstrated that vitamin D supplementation improved handgrip strength in subjects with dosages > 1000 IU/day, treatment duration >3 months, and baseline vitamin D < 30 ng/mL [151]. More recently, an RCT of patients with decompensated LC and vitamin D insufficiency demonstrated that vitamin D supplementation improved SMI and handgrip strength [152].

#### 3.3.3. Pharmacological Treatment

Osteoporosis is caused by impaired bone homeostasis due to an imbalance between bone remodeling and resorption. Pharmacological treatment for osteoporosis includes anti-resorptive (e.g., bisphosphonates and denosumab), anabolic (e.g., teriparatide), anti-sclerostin (e.g., romosozumab), and hormonal agents (e.g., hormone replacement therapy and selective estrogen-receptor modulators) [153]. The receptor activator of nuclear factor kappa-B ligand (RANKL), which is expressed on osteoblasts and osteocytes, binds to the receptor RANK on osteoclasts and promotes differentiation and activation of osteoclasts, leading to bone resorption [154]. Osteoprotegerin (OPG), a soluble decoy receptor for RANKL, prevents the interaction between RANK and RANKL, and thus suppresses osteoclastogenesis and bone loss. Denosumab is a human monoclonal antibody for RANKL and mimics the endogenous effect of OPG, thereby inhibiting bone resorption and remodeling. Our study of CLD patients with osteoporosis revealed that denosumab increased BMD, suppressed bone turnover, and improved a bone quality marker after 12 months of treatment [155]. Meanwhile, RANK is expressed in skeletal muscle, and muscle-specific RANK deletion was protective against denervation-induced loss of muscle force in mice [156]. A recent study of postmenopausal women with osteoporosis revealed that denosumab improved physical performance and skeletal muscle strength as compared to bisphosphonates [157]. A pooled analysis demonstrated that denosumab reduces the risk of falling by 20% [158]. These results suggest that denosumab has a potential dual effect on osteoporosis and sarcopenia. As described above, myostatin is a potent negative regulator of muscle protein synthesis, and there have been clinical studies conducted on myostatin-targeted treatment [159,160]. ACE-031, a soluble form of ActRIIB, binds ActRIIB ligands including myostatin, GDF11, and activin by acting as a decoy receptor, which promotes muscle growth [159]. A double-blind, placebo-controlled, phase 1 study revealed that ACE-031 treatment was well-tolerated, increased lean muscle mass and muscle volume, and improved a bone turnover marker in postmenopausal women [159]. In a multicenter, phase 2 study of patients aged ≥75 years with a history of fall, administration of humanized myostatin antibody LY2495655 (LY) increased lean muscle mass and improved several performance-based measures [158]. However, targeting a broad spectrum of TGF-β family members can induce adverse effects, such as telangiectasia and epistaxis [161,162]. In addition, myostatin is expressed in cardiac tissue, and sustained myostatin inhibition might cause cardiomyopathy [163]. A recent in vivo study focused on another endogenous antagonist, follistatin-like 3 (FSTL3), which has a more restricted binding profile for TGF-β family ligands. The administration of FSTL3 Fc-fusion protein promoted muscle fiber hypertrophy and increased muscle mass [162]. Accordingly, molecular target treatments have either been developed or are under investigation for osteoporosis and sarcopenia. However, further studies are needed to confirm the long-term effects and safety of such treatments.

## Figures and Tables

**Table 1:** Reference values for reduced skeletal muscle mass and strength, and physical performance in the JSH, EWGSOP, and AWGS criteria.

	Measurement Method	JSH	EWGSOP	EWGSOP2	AWGS	AWGS 2019
Muscle mass	DEXA		M: YAM − 2SD	M: 7.0 kg/m^2^	M: 7.0 kg/m^2^	M: 7.0 kg/m^2^
			F: YAM − 2SD	F: 5.5 kg/m^2^	F: 5.4 kg/m^2^	F: 5.4 kg/m^2^
	BIA	M: 7.0 kg/m^2^			M: 7.0 kg/m^2^	M: 7.0 kg/m^2^
		F: 5.7 kg/m^2^			F: 5.7 kg/m^2^	F: 5.7 kg/m^2^
	CT (L3 level)	M: 42 cm^2^/m^2^				
		F: 38 cm^2^/m^2^				
Muscle strength or Physical performance	Handgrip strength	M: 26 kg	M: 30kg	M: 27 kg	M: 26 kg	M: 28 kg
		F: 18 kg	F: 20kg	F: 16 kg	F: 18 kg	F: 18 kg
	Chair stand test (5-time)			15 s		12 s
	Gait speed		0.8 m/s	0.8 m/s	0.8 m/s	1.0 m/s
	SPPB		8 points	8 points	9 points	9 points

Abbreviations: AWGS, Asian Working Group for Sarcopenia; BIA, bioelectrical impedance analysis; CT, computed tomography; DEXA, dual-energy X-ray absorptiometry; EWGSOP, European Working Group on Sarcopenia in Older People; JSH, Japan Society of Hepatology; SD, standard deviation; SPPB, Short Physical Performance Battery; TUG, Timed-Up and Go test; YAM, young adult mean.

**Table 2:** Representative previous studies on osteosarcopenia in patients with chronic liver disease.

Authors (Year, Country) [Reference]	Patients Characteristics	Prevalence of Osteosarcopenia	Diagnostic Method for Osteosarcopenia (Criteria for Sarcopenia)	Main Findings
Hayashi et al. (2018, Japan) [85]	112 patients with CLD (LC, 36.0%)	7.1%	DEXA and BIA (JSH)	Sarcopenia and LC were significantly associated with the BMD. Sarcopenia (OR, 6.16) and LC (OR, 15.8) were independent risk factors for osteoporosis.
Bering et al. (2018, Brazil) [86]	104 patients with CHC	―	DEXA (EWGSOP)	Low BMD, low muscle strength, pre-sarcopenia, and sarcopenia were noticed in 34.6%, 27.9%, 14.4%, and 8.7% of subjects, respectively. Appendicular skeletal muscle mass was an independent predictor of BMD. Sarcopenia was independently related to bone mineral content.
Saeki et al. (2020, Japan) [21]	117 patients with PBC (LC, 9.4%)	15.4%	DEXA and BIA (JSH)	The SMI and handgrip strength were significantly correlated with the BMD. Patients with osteosarcopenia had a higher prevalence of vertebral fracture (55.6%) than those without both sarcopenia and osteoporosis (6.7%).
Saeki et al. (2020, Japan) [22]	291 patients with CLD (LC, 51.9%)	LC 20.5% Non-LC 12.9%	DEXA and BIA (JSH)	Frailty was an independent risk factor associated with osteosarcopenia (OR, 9.837), and vice versa (OR, 10.069). The prevalence of frailty and vertebral fracture was significantly higher in patients with osteosarcopenia than in those without osteosarcopenia (79.6% vs. 17.4% and 59.2% vs. 20.2%, respectively).

Abbreviations: BIA, bioelectrical impedance analysis; BMD, bone mineral density; CHC, chronic hepatitis C; CLD, chronic liver disease; DEXA, dual-energy X-ray absorptiometry; EWGSOP, European Working Group on Sarcopenia in Older People; JSH, Japan Society of Hepatology; LC, liver cirrhosis; OR, odds ratio; PBC, primary biliary cholangitis; SMI, skeletal muscle mass index.

## Data Availability

Not applicable.
